# Reactive oxygen species induced structural alterations of substance P

**DOI:** 10.1155/S096293519200053X

**Published:** 1992

**Authors:** Y. T. Konttinen, P. Kemppinen, M. Segerberg, T. Sorsa, H. Saari, M. Hukkanen

**Affiliations:** 1 Department of Anatomy Siltavuorenpenger 20 A Helsinki SF-O0170 Finland; 2 Department of Medical Chemistry University of Helsinki Siltavuorenpenger 10 Helsinki SF-O0170 Finland; 3 Department of Periodontology University of Helsinki Mannerheimintie 172 Helsinki SF-00170 Finland; 4 Department of Histochemistry Royal Postgraduate Medical School Du Cane Road London W12 ONN UK

## Abstract

Substance **P** (SP_1-11_) was exposed to a continuous flux of superoxide (${\text{O}}_{2}^{-}$) or hydroxyl radicals (^.^OH) in a hypoxanthine (HX)/xanthine oxidase (86 mU) system in the presence of 1 mM deferoxamine and 40 mM D-mannitol or 50 μM FeCI_3_. 6H_2_O and 50 μM EDTA, respectively. ${\text{O}}_{2}^{-}$ caused fragmentation between the Phe^7^ and Phe^8^, whereas ^.^OH induced cleavage also between the Phe^8^ and Gly^9^. Reactive oxygen species H_2_O_2_ and HCIO did not cause fragmentation, but modification of the amino acid side chains and/or aggregation with altered hydrophobicity in reverse phase high performance liquid chromatography compared to native SP_1-11_. Furthermore, exposure of SP_1-11_ to phorbol myristate acetate preactivated neutrophils resuited in products similar to those observed upon exposure to superoxide or hydroxyl radicals in a cell-free HX/xanthine oxidase system. This study suggests that, in contrast to rigid proteins, fragmentation is relatively easily induced in a small peptide like SP_1-11_, perhaps due to strain on the peptide and t-carbon bonds caused by the movable, random coil configuration acquired by SP_1-11_ in an aqueous solution. Oxidative modification might modulate paracrine actions of SP_1-11_ at site of inflammation.


### Research Paper

Mediators of Inflammation, 1, 355–360 (1992)

**SUBSTANCE P ($SP_{1-11}$) was exposed to a continuous flux of superoxide ($O_2^-$) or hydroxyl radicals ($^{\bullet}OH$) in a hypoxanthine (HX)/xanthine oxidase (86 mU) system in the presence of 1 mM deferoxamine and 40 mM D-mannitol or 50 μM $FeCl_3 \cdot 6H_2O$ and 50 μM EDTA, respectively. $O_2^-$ caused fragmentation between the $Phe^7$ and $Phe^8$, whereas $^{\bullet}OH$ induced cleavage also between the $Phe^8$ and $Gly^9$. Reactive oxygen species $H_2O_2$ and HClO did not cause fragmentation, but modification of the amino acid side chains and/or aggregation with altered hydrophobicity in reverse phase high performance liquid chromatography compared to native $SP_{1-11}$. Furthermore, exposure of $SP_{1-11}$ to phorbol myristate acetate preactivated neutrophils resulted in products similar to those observed upon exposure to superoxide or hydroxyl radicals in a cell-free HX/xanthine oxidase system. This study suggests that, in contrast to rigid proteins, fragmentation is relatively easily induced in a small peptide like $SP_{1-11}$, perhaps due to strain on the peptide and α-carbon bonds caused by the movable, random coil configuration acquired by $SP_{1-11}$ in an aqueous solution. Oxidative modification might modulate paracrine actions of $SP_{1-11}$ at site of inflammation.**

**Key words:** Reactive oxygen species, Substance P

### Reactive oxygen species induced structural alterations of substance P

**Y. T. Konttinen,[1,CA] P. Kemppinen,[1] M. Segerberg,[1] T. Sorsa,[2,3] H. Saari[1] and M. Hukkanen[4]**

[1]Department of Anatomy, Siltavuorenpenger 20 A, SF-00170 Helsinki, Finland;
[2]Department of Medical Chemistry, University of Helsinki, Siltavuorenpenger 10, SF-00170 Helsinki, Finland;
[3]Department of Periodontology, University of Helsinki, Mannerheimintie 172, SF-00170 Helsinki, Finland;
[4]Department of Histochemistry, Royal Postgraduate Medical School, Du Cane Road, London W12 0NN, UK

[CA] Corresponding Author

### Introduction

Substance P ($SP_{1-11}$) is an undecapeptide, which consists of Arg-Pro-Lys-Pro-Gln-Gln-Phe-Phe-Gly-Leu-Met-$NH_2$.[1] $SP_{1-11}$ has been localized to small unmyelinated, slowly conducting C-type polymodal nociceptors.[2] In addition to its possible role in pain transmission in the dorsal horn of the spinal cord,[3] it has been implicated in neurogenic inflammation.[4] Local release by axon reflex as a result of antedromic conduction causes wheal and flare,[4] but various paracrine effects such as synthesis and secretion of type I matrix metalloproteinase (MMP-1 or 'fibroblast-type' interstitial collagenase) and prostaglandin $E_2$ ($PGE_2$) by synoviocytes,[5] and of interleukin-1β and tumour necrosis factor-α by monocytes, have also been described.[6] In addition to its role in vasoregulation and cytokine and proteinase secretion, substance P can activate polymorphonuclear neutrophils (PMN) and monocyte/macrophages to produce superoxide ($O_2^-$) via the NADPH oxidase pathway.[7,8] In many inflammatory diseases hypoxia-reperfusion syndrome is another and possibly more important source of oxygen derived free radicals (ODFR).[9] During ischaemia xanthine dehydrogenase is proteolytically cleaved to xanthine oxidase by an enzyme activated by $Ca^{2+}$ efflux from mitochondria. At the same time, chemical energy from adenosine triphosphate (ATP) is utilized and hypoxanthine (HX) is produced. During reperfusion xanthine oxidase will catalyse the conversion of HX to xanthine and further to uric acid (Equation 1, see below), with bimolecular oxygen acting as an electron acceptor in both reactions. It seems, therefore, that $SP_{1-11}$, released at a site of inflammation, will be exposed not only to degradative enzymes but also to various reactive oxygen species. This prompted us to study the possible effect of such compounds on substance P *in vitro*.

### Materials and Methods

*Preparation of synthetic substance P:* Synthetic substance P was first purchased from Sigma Chemical Company (St Louis, MO, USA) or Cambridge Research Biochemicals (Cambridge, UK). Because relatively large amounts were needed, substance P was also synthesized according to the solid-phase method with Applied Biosystems (Foster City, CA, USA) 430A peptide synthesizer using tBoc chemistry and p-methyl-BHA-resin.

© 1992 Rapid Communications of Oxford Ltd

*Production of oxygen-derived free radicals:* Synthetic soluble substance P (1 mg/ml) in RPMI-1640 (Gibco) was exposed to $O_2^-$ or hydroxyl radical ($^{\cdot}OH$) produced in a hypoxanthine/xanthine oxidase (EC 1.2.3.22) system in the presence of 1 mM deferoxamine (Desferal®, Ciba Pharmaceutical Co) and 40 mM D-mannitol or 50 $\mu$M $FeCl_3 \cdot 6H_2O$ (Mallinckrodt, Paris, KY, USA) and 50 $\mu$M ethylenediaminetetra-acetic acid (EDTA) (Sigma), respectively, as described in detail elsewhere.[10,11] HX was added in excess (up to a 10 mM final concentration). Deferoxamine chelates iron by occupying all of its four coordination sites and iron is thus altered to a catalytically inactive form,[12] whereas mannitol is an effective hydroxyl radical scavenger.[13] In the presence of these reagents, alterations observed are caused by $O_2^-$, although $H_2O_2$ is also formed in a so-called one-electron auto-oxidation or dismutation (Equation 2). Iron was added as iron(III) ions, so that the reaction would start immediately after addition of xanthine oxidase: in this case, iron(III) ions are first reduced to iron(II) ions (Equation 3). Iron(II) ions can then act as an electron donor and catalyse the Fenton reaction, in which $H_2O_2$ is converted to hydroxyl ion and hydroxyl radical (Equation 4). The combination of Equations 3 and 4 is the so-called Haber–Weiss reaction (Equation 5). When $^{\cdot}OH$ was produced, EDTA was added in addition to iron(III) ions to increase the effective concentration of iron, which is difficult to dissolve in water based buffers; iron complexed with EDTA is redox reactive. All reactions were started by adding xanthine oxidase, performed under constant stirring at 22°C and stopped by addition of 10 $\mu$g/ml superoxide dismutase (EC 1.15.1.1) and catalase (EC 1.11.1.6). In addition to ODFR, $SP_{1-11}$ was also exposed to reactive oxygen species $H_2O_2$ (1 and 0.1 mM) or HClO (added as calcium salt, 1 and 0.1 mM) The HClO reaction was stopped by L-methionine as scavenger,[14] which at the end of the incubation was added to 10 mM final concentration.

$$(1)\quad HX \xrightarrow{XAO} \text{xanthine} \xrightarrow{XAO} \text{urate}$$

$$(2)\quad O_2^- + O_2^- + 2H^+ \longrightarrow H_2O_2 + O_2$$

$$(3)\quad Fe^{3+} + O_2^- \longrightarrow Fe^{2+} + O_2$$

$$(4)\quad Fe^{2+} + H_2O_2 \longrightarrow Fe^{3+} + OH^- + {}^{\cdot}OH$$

$$(5)\quad H_2O_2 + O_2^- \longrightarrow O_2 + OH^- + {}^{\cdot}OH$$

Samples from substance P exposed to $O_2^-$, $^{\cdot}OH$, $H_2O_2$ or HClO were subjected to reverse phase high performance liquid chromatography (HPLC).

*Exposure of substance P to phorbol myristate acetate preactivated human neutrophils:* Polymorphonuclear neutrophilic leukocytes were separated from venous heparinized blood obtained from three healthy volunteers by using Ficoll-Hypaque (Pharmacia LKB Biotechnology, Uppsala, Sweden) density gradient centrifugation (400 × *g*, 45 min) followed by sedimentation of red blood cells in 1.5% dextran (T500, Pharmacia Fine Chemicals, Uppsala, Sweden) as described in detail elsewhere.[15] Purified neutrophils ($20 \times 10^6$/2 ml RPMI-1640) were activated with 50 $\mu$g/ml of phorbol 12-myristate 13-acetate (PMA; Sigma) at 37°C for 30 min.[15] Even though the PMA concentration we use[15] is rather high, it does not cause cell lysis so that neutrophil viability was >98% as assessed by trypan blue exclusion. After PMA preactivation, the cells were separated by spinning the neutrophils at 1000 × *g* for 5 min and resuspended in 1 ml RPMI-1640. 1 mg of substance P was added to each tube and the samples were incubated at +37°C for the time periods indicated and 100 $\mu$l aliquots were taken for analysis. Samples were spun at 1000 × *g* for 5 min and 50 $\mu$l of the supernatent was frozen immediately to be analysed later by HPLC.

*High performance liquid chromatography:* Separation of synthetic substance P exposed to different reactive oxygen species as described above was done by high performance liquid chromatography (HPLC). Samples were loaded onto a Nova-Pak™ C18 4 $\mu$m column (silica, 3.9 mm × 15 cm, pore diameter 600 nm) protected by Guard-Pak™ precolumn module and Bondapak C18 inserts using a WISP™ model 710 B sample processor. After a 2 min loading period a 60 min elution was done with a linear 1 ml/min gradient (0–48%) with 50 mM $NaH_2PO_4/H_3PO_4$, pH 3.0, as buffer and 60% $CH_3CN$ in buffer as eluent. Two model 510 high performance pumps were controlled with a model 680 automated gradient controller. Absorbance was read at 214 nm with a model 441 on-line detector and recorded using a model 730 printer/plotter/integrator data module. All HPLC equipment was from Waters Associates, Milford, MA, USA. To identify oxidative substance P degradation products by their chromatographic motility, synthetic $SP_{1-4}$, $SP_{1-8}$, $SP_{7-11}$, $SP_{8-11}$, $SP_{9-11}$ and substance P free acid, all from Sigma, were also studied similarly.

### Results

Exposure to a continuous flux of superoxide caused formation of two major oxidation products, which had retention times similar to $SP_{8-11}$ (31.90 min) and $SP_{1-7}$ (27.68 min) and probably represent oxidative fragmentation of substance $P_{1-11}$

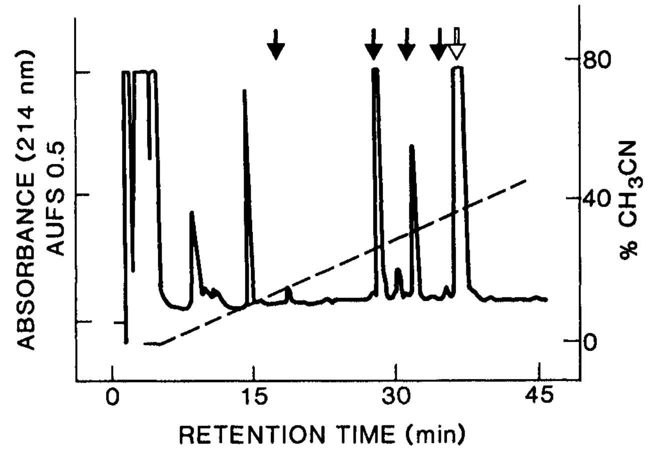

FIG. 1. HPLC chromatogram of substance $P_{1-11}$ exposed to hydroxyl radical for 2 h. Synthetic substance P (1 mg/ml) in RPMI-1640 was under continuous stirring incubated at +22°C in the presence of an excess of hypoxanthine, 85 mU xanthine oxidase, 50 μM $FeCl_3 \cdot H_2O$ and 50 μM EDTA. A 100 μl aliquot was recovered and the reaction was stopped by adding superoxide dismutase and catalase to a 1 μg/ml final concentration. A 50 μl aliquot was then run over a linear 60 min 0–48% 1 ml/min $CH_3CN$ gradient with 50 mM $NaH_2PO_4/H_3PO_4$, pH 3.0, as a buffer and 60% $CH_3CN$ in buffer as eluent. The white arrow shows the native substance $P_{1-11}$ (retention time 36.38 min) and black arrows show the major oxidation products (peaks with retention times 31.95 min corresponding to $SP_{8-11}$ and 28.01 min corresponding to $SP_{1-7}$) and minor oxidation products (peaks with retention times 18.33 min corresponding to $SP_{9-11}$ and 35.78 min corresponding to $SP_{1-8}$). All other peaks represent components of the incubation medium or constant injection artefacts, which did not change over time.

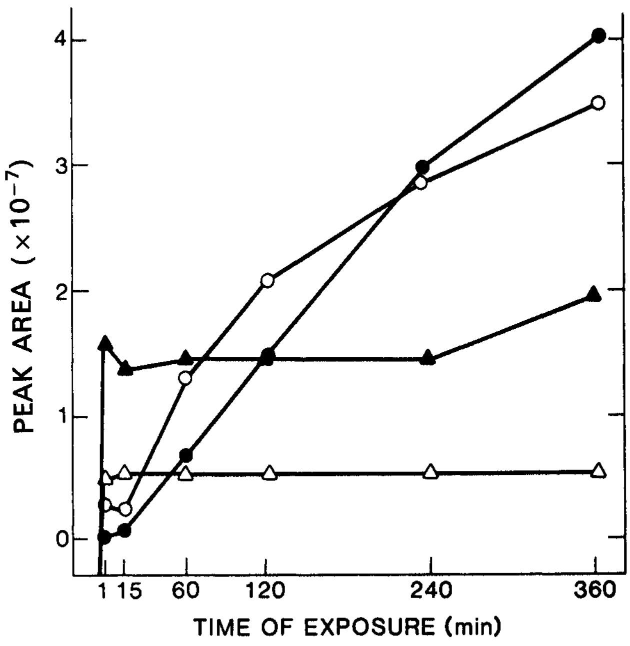

FIG. 2. A time course experiment on the formation of the major oxidation products (peak with retention time 31.95 min corresponding to $SP_{8-11}$ has been marked with circles and a peak with retention time 28.01 min corresponding to $SP_{1-7}$ has been marked with triangles). Oxygen derived free radical flux was produced in the hypoxanthine/xanthine oxidase (85 mU) system in the presence of 1 mM deferoxamine and 40 mM D-mannitol or 50 μM $FeCl_3 \cdot H_2O$ and 50 μM EDTA to produce superoxide (open symbols) or hydroxyl radicals (closed symbols), respectively.

between the two phenylalanine residues at positions 7 and 8. Exposure to hydroxyl radicals produced similar fragments, but, in addition, two minor oxidation products with retention times 31.17 min and 18.33 min were produced (Figure 1). One of these had a retention time similar to $SP_{9-11}$ (18.33 min) and although $SP_{1-8}$ was not available for chromatographic analysis, these two minor hydroxyl radical oxidation products could, by deduction, represent an additional cleavage site between phenylalanine and glycine residues at positions 8 and 9.

If instead of ODFR, $SP_{1-11}$ was exposed to reactive oxygen species $H_2O_2$ (1 and 0.1 mM) or HClO (added as calcium salt, 1 and 0.1 mM), fragmentation was not observed (only one reaction product was produced), but rather modification of the amino acid side chains and/or aggregation: $H_2O_2$ and HClO induced the formation of oxidation products with retention times 33.13 min and 32.04 min, respectively.

Oxidative modification of synthetic soluble substance P in aqueous solution was time-dependent. Fragmentation caused by superoxide and hydroxyl radical seemed to have a somewhat similar temporal profile (Figure 2). Oxidative modification induced by hydrogen peroxide was a relatively slow process (32.04 min product) compared to the almost immediate effect seen when

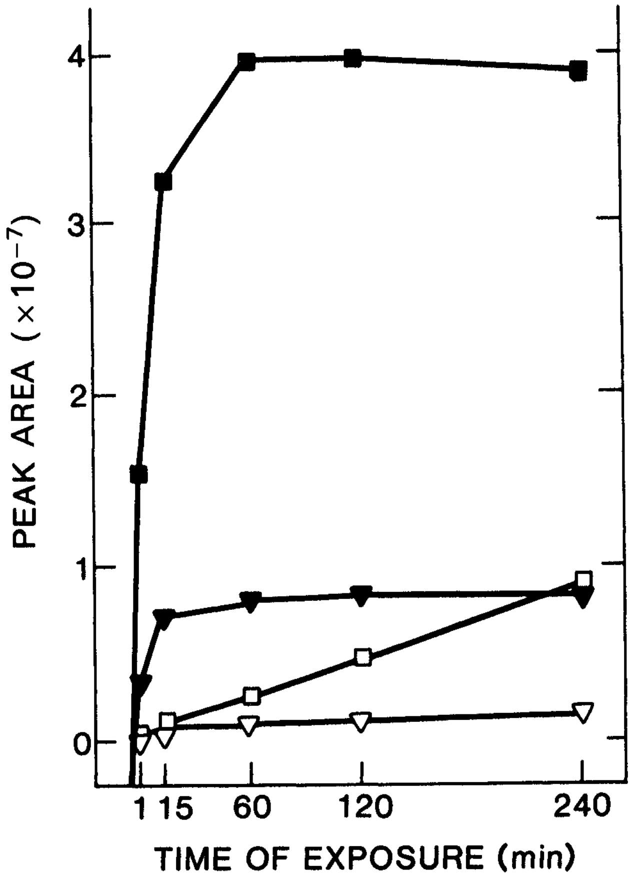

FIG. 3. A time course experiment on the oxidative modification caused by 0.1 mM (open symbols) or 1 mM (closed symbols) hypochlorous acid (squares) or hydrogen peroxide (inverted triangles).

**Table 1.** Effect of PMA preactivated neutrophils on synthetic substance P. Human peripheral blood neutrophils (PMN) were isolated and preactivated by incubation in 50 $\mu$g/ml PMA at 37°C for 30 min. These preactivated cells were resuspended in 1 ml RPMI-1640 medium to which 1 mg/ml substance P was added. 100 $\mu$l aliquots were recovered at the times indicated and the cell free supernatant was analysed for substance P and its oxidation products using high performance liquid chromatography (HPLC) in peak area mode[a]

| Peak[b] | Incubation time with PMA preactivated neutrophils | | | | | |
|---|---|---|---|---|---|---|
| | 1 min | 5 min | 15 min | 30 min | 1 h | 2 h |
| 31.60 min | 216 | 980 | 1882 | 1823 | 1472 | 1012 |
| 28.53 min | 263 | 554 | 204 | 1728 | 1916 | 2256 |
| 18.45 | 699 | 2641 | 7563 | 9753 | 10792 | 13165 |

[a] The areas of the peaks are expressed in arbitrary units calculated by the integrator ($\times 10^{-3}$).
[b] Peaks are defined here by their retention time in HPLC.

hypochlorous acid was used (33.13 min product) Figure 3).

Synthetic $SP_{1-11}$ in aqueous solution was rapidly modified by PMA preactivated neutrophils. Peaks with retention times corresponding to those of $SP_{8-11}$, $SP_{1-7}$ and $SP_{9-11}$ appeared in the supernatant, whereas the peak corresponding to the minor $SP_{1-8}$ peak produced by ˙OH in the HX/xanthine oxidase system was negligible (one representative experiment is shown in Table 1). In addition, peaks eluting at approximately 38.7 min, 40.2 min and 41.3 min were also found in the substance P–neutrophil supernatant aliquots (not shown).

### Discussion

These results obtained using cell free conditions show that $SP_{1-11}$ can be fragmented by ODFR, both $O_2^-$ and ˙OH. Oxidative modifications of various free amino acids and proteins have received a lot of attention,[16] but observations on the effect of ODFR on substance P have not been published. The effect of ODFR on various amino acids cannot be extrapolated to proteins or peptides due to their often complex secondary and tertiary structure, which will affect the sites accessible for the initial attack and the subsequent secondary effects such as intramolecular charge transfer reactions. Proteins, perhaps due to the usually relatively rigid structure possibly enforced by intra- and interchain disulphide bonds, are usually not fragmented by ODFR, although such an exposure may make them susceptible or mark them for a subsequent proteolytic attack.[17] According to our finding, however, a small peptide, substance P, was fragmented by both $O_2^-$ and ˙OH. Substance P in aqueous phase acquires a mobile random coil configuration,[18] with nonregular, nonrepeating dihedral angles of the peptide backbone with or without equilibrium between various random coil conformers. This may strain the peptide or α-carbon bonds and may contribute to their cleavage.

Some amino acids are more susceptible to oxidative damage than others.[19] Phenylalanine containing an aromatic benzene ring is easily oxidized. Exposure of phenylalanine to ˙OH will cause hydroxylation to the *p*-, *m*- and in particular *o*-position. Two such radicals can join together to give a dimer, that can lose water to form biphenyl.[20] The close apposition of phenylalanine residues at positions 7 and 8 in a flexible random coil $SP_{1-11}$ might favour the formation of such biphenyls, which could cause a sudden change, a nick, in the stereoconfiguration. That non-enzymatic, conformational changes can cleave even peptide bonds is suggested by the action of catalytic antibodies.[21] An antibody to vasoactive intestinal peptide (VIP) can cause cleavage of a peptide bond in full-length $VIP_{1-28}$. Interestingly, and against expectations, the scissile bond was not part of the antigenic determinant involved in the high affinity antigen–antibody binding. Therefore, the relatively strong interactive forces involved in binding of the catalytic antibody to $VIP_{1-28}$ are unlikely to be directly responsible for the peptide bond hydrolysis. Competitive binding studies using various synthetic VIP peptide fragments were used to map the antibody binding epitope to amino acid residues 22–28. This binding was able to cause a cleavage at a distant scissile $Glu^{16}$–$Met^{17}$ bond, perhaps via conformational changes induced by binding of the catalytic antibody.

Neuropeptide peptide bonds are cleaved by various well described exo- and endopeptidases. Actually, ODFR usually lead to formation of α-carbon centred radicals and, in the presence of oxygen, peroxyl radicals, which decompose to fragment the polypeptide chain at the α-carbon rather than at peptide bond.[14] Accordingly, the cleavage sites observed in the present study, i.e. between the phenylalanine residues at positions 7 and 8 and, with ˙OH also between the phenylalanine and glycine at positions 8 and 9, may be located at the α-carbon rather than the peptide bond. Obviously, the hypothesis about the fragments of substance P generated by oxygen radical exposure has to be validated in future studies with appropriate nuclear magnetic resonance and mass spectroscopy studies allowing the identification of the structure of the generated metabolites.

In contrast to $O_2^-$ and ˙OH, $H_2O_2$ is, by definition, not a radical: it does not contain unpaired electrons i.e. electrons occupying an atomic or molecular orbital by itself. It is not particularly reactive either, compared to ODFR. On the other hand, $H_2O_2$, like water, as a small, uncharged polar molecule, has a very high permeability coefficient compared to e.g. charged $O_2^-$. It can therefore, pass through lipid biomembranes. It can also, according to Equation 4 (see above), lead to the formation of the highly reactive hydroxyl radical in the presence of transition metal ions like iron and copper. In the present study, when $H_2O_2$ was added to an aqueous solution, it did not cause fragmentation of $SP_{1-11}$, but instead a slowly progressive structural modification, which was reflected in altered hydrophobicity of the reaction product in reverse phase HPLC. In contrast, HClO caused a rapid modification of $SP_{1-11}$, which again was reflected in altered hydrophobicity. It was noteworthy that the HClO-induced product had a different retention time than the $H_2O_2$-induced oxidation product. Although not studied in structural detail, it is likely that HClO caused conversion of the methionine residue at position 11 to a corresponding methionine sulphoxide (MetSO) or perhaps even to a methionine sulphone ($MetSO_2$).[22] Also this reaction pathway would seem to be of potential relevance *in vivo* in inflammation, because under such circumstances HClO is formed from $H_2O_2$ by myeloperoxidase in the presence of chloride ion. Chloride ion is one of the most common ions in the extracellular tissue fluid and myeloperoxidase is stored in and, upon activation, released from the primary or azurophil granules of the PMN.[23]

PMA is a direct activator of the protein kinase C and will, by phosphorylation to serine and threonine residues of some as yet mostly unknown target proteins, activate the cell membrane NADPH oxidase to produce $O_2^-$. This will spontaneously dismutate to $H_2O_2$ at a rate of $2 \times 10^{-5}$ $Ms^{-1}$. In the presence of trace amounts of iron, always present in 'average' reaction mixtures without added metal, usually at about 1 $\mu$M as assessed by atomic absorption analysis,[20] hydroxyl radicals also are likely to be formed. The interpretation of PMA preactivated neutrophil experiments is complicated by cellular uptake and by simultaneous activation of neutrophil-mediated exocytosis as was suggested by the presence of peaks other than those produced in the cell free HX/xanthine oxidase system. Neutrophils contain in their primary granules cathepsin G, which, according to the bond specificity is able to cleave substance P. In addition, neutrophils contain an integral membrane protein known as neutral endopeptidase EC 3.4.24.11, also known as the common acute lymphoblastic leukaemia associated antigen CALLA and as enkephalinase, which is also able to cleave substance P.[24,25] Therefore, even if untreated neutrophils would cause substance P degradation or superoxide scavenger superoxide dismutase would not be able to inhibit substance P degradation, it is not possible to exclude the role of oxidative degradation because there are alternative and complementary degradative pathways. However, these results suggest that substance P exposed to PMA preactivated neutrophils is modified as if exposed to superoxide or hydroxyl radicals in a cell free HX/xanthine oxidase system *in vitro*.

It therefore seems that various ODFR and reactive oxygen species can cause oxidant specific and time-dependent modification of synthetic $SP_{1-11}$ in aqueous phase by inducing fragmentation, modification of side chains and aggregation. According to the present findings, it seems likely that oxidative modification also has to be taken into consideration when the paracrine actions of $SP_{1-11}$ in inflammatory diseases and processes are regarded: the distance from the axon terminal to the potential site of action may at the paracrine site of action be more than 10 000 longer than in the more concealed synaptic spaces.

### References

1. Chang MM, Leeman SE, Niall HD. Amino acid sequence of substance P. *Nature* 1971; **232:** 86–87.
2. Buck SH, Walsh JH, Yamamura HI, Burks TF. Neuropeptides in sensory neurons. *Life Sci* 1982; **30:** 1857–1866.
3. Fields HL. Introduction. In: Fields HL, ed. *Pain Syndromes in Neurology.* London: Butterworths 1990; 1–18.
4. Lembeck F, Holzer P. Substance P as neurogenic mediator of antidromic vasodilation and neurogenic plasma extravasation. *Naunyn-Schmiedeberg's Arch Pharmacol* 1979; **310:** 175–183.
5. Lotz M, Carson DA, Vaughan JH. Substance P activation of rheumatoid synoviocytes: neural pathway in pathogenesis of arthritis. *Science* 1987; **235:** 893–895.
6. Lotz M, Vaughan JH, Carson DA. Effect of neuropeptides on production of inflammatory cytokines by human monocytes. *Science* 1988; **241:** 1218–1221.
7. Hartung HP, Toyka KV. Activation of macrophages by substance P: induction of oxidative burst and thromboxane release. *Eur J Pharmacol* 1983; **89:** 301–305.
8. Serra MC, Bazzoni F, Bianca VD, Greskowiak M, Rossi F. Activation of human neutrophils by substance P. Effect on oxidative metabolism, exocytosis, cytosolic $Ca^{++}$ concentration and inositol phosphate formation. *J Immunol* 1988; **141:** 2118–2124.
9. Blake DR, Merry P, Unsworth J, *et al.* Hypoxic-reperfusion injury in the inflamed joint. *Lancet* 1989; **i:** 289–293.
10. Sorsa T, Saari H, Konttinen YT, Uitto V-J, Lindy S. Human neutrophil collagenase and oxygen derived free radicals. *N Engl J Med* 1989; **321:** 327–328.
11. Saari H, Suomalainen K, Lindy O, Konttinen YT, Sorsa T. Activation of latent human neutrophil collagenase by reactive oxygen species and serine proteases. *Biochem Biophys Res Commun* 1990; **171:** 979–987.
12. Graf E, Mahoney JR, Bryant RG, Eaton JW. Iron catalyzed hydroxyl radical formation. Stringent requirement for free iron coordination site. *J Biol Chem* 1984; **259:** 3620–3624.
13. Weiss SJ, Ragiani S. Neutrophils degrade subendothelial matrices in the presence of alpha-1-proteinase inhibitor. Cooperative use of lysomal proteinases and oxygen metabolites. *J Clin Invest* 1984; **73;** 1297–1303.
14. Garrison WM, Jayko ME, Bennett W. Radiation-induced oxidation of protein in aqueous solution. *Radiat Res* 1962; **16:** 483–502.
15. Konttinen YT, Lindy O, Kemppinen P, *et al.* Collagenase reserves in polymorphonuclear neutrophil leukocytes from synovial fluid and peripheral blood of patients with rheumatoid arthritis. *Matrix* 1991; **11:** 296–301.
16. Davies KJA. Intracellular proteolytic systems may function as secondary antioxidant defences: an hypothesis. *Free Radic Biol Med* 1987; **2:** 155–173.
17. Davies KJA. Protein damage and degradation by oxygen radicals. I. General aspects. *J Biol Chem* 1987; **262:** 9895–9901.
18. Mehlis B, Rueger M, Becker M, Bienert M, Niedrich H, Oehme P. Circular dichroism studies of substance P and its C-terminal sequences. CD spectra in aqueous solution and effects of hydrogen ion concentration. *Int J Peptide Prot Res* 1980; **15:** 20–28.
19. Davies KJA, Delasignore ME, Lin SW. Protein damage and degradation by oxygen radicals. II. Modification of amino acids. *J Biol Chem* 1987; **262:** 9902–9907.
20. Halliwell B, Gutteridge JMC, eds. *Free Radicals in Biology and Medicine.* 2nd edn. Oxford: Clarendon Press 1989.

21. Paul S, Volle DJ, Powell MJ, Massey RJ. Site specifity of a catalytic vasoactive intestinal peptide antibody. An inhibitory vasoactive intestinal peptide subsequence distant from the scissile. *J Biol Chem* 1990; **265:** 11910–11913.
22. Swaim MW, Pizzo SV. Methionine sulfoxide and the oxidative regulation of plasma proteinase inhibitors. *J Leukoc Biol* 1988; **43:** 365–379.
23. Sepe SM, Clark RA. Oxidant membrane injury by the neutrophil myeloperoxidase system. I. Characterization of a liposome model and injury by myeloperoxidase, hydrogen peroxide and halides. *J Immunol* 1985; **134:** 1888–1898.
24. Erdös EG, Wagner B, Harbury CB, Painter RG, Skidgel RA, Fa XG. Down-regulation and inactivation of neutral endopeptidase 24.11 (enkephalinase) in human neutrophils. *J Biol Chem* 1989; **264:** 14519–14523.
25. Shipp MA, Stefano GB, Switzer SN, Griffin JD, Reinherz EL. CD10 (CALLA)/neutral endopeptidase 24.11 modulates inflammatory peptide-induced changes in neutrophil morphology, migration, and adhesion proteins and is itself regulated by neutrophil activation. *Blood* 1991; **78:** 1834–1841.

ACKNOWLEDGEMENTS. This project was supported in part by the Finnish Academy, the Emil Aaltonen Foundation, the Finska Läkaresällskapet and the Perklen Foundation. We (Y.T.K., P.K., M.S.) are grateful to our host Professor Robert J. Winchester for placing the facilities of his institution at our disposal during our research sabbatical to Hospital for Joint Diseases, Orthopaedic Institute, New York, NY, USA.

**Received 24 June 1992; accepted in revised form 18 August 1992**
